# Supplementary material for: Synchronization of spin-driven limit cycle oscillators optically levitated in vacuum
Source: Nat Commun. 2023 Sep 6;14:5441. doi: 10.1038/s41467-023-41129-5 (PMC10482900; doi:10.1038/s41467-023-41129-5)
Supplement: Supplementary file 1 — Supplementary Information [file 41467_2023_41129_MOESM1_ESM.pdf]

Synchronization of spin-driven limit cycle oscillators optically levitated in vacuum : Supplementary Information

CONTENTS

I. Physical System 1

II. Model And Calculations 2

III. General Theory For Linear Forces 3
A. Perturbation Theory And Spectral Decomposition 4
B. Linearly Conservative And Linearly Non-Conservative Systems 7
C. Normal Modes 7
D. Quasi-Modes, Complex Eigenvalues 8

IV. Spin-driven Oscillators In The Linear Regime 9
A. Stiffness Matrix 9
B. Hydrodynamic Resistance 12
C. Stochastic Motion 12
1. Single Spin Oscillators 13
2. Coupled Spin-driven Oscillators 14

V. Above Threshold Behaviour 15
A. Limit Cycle Formation 15
B. Definition Of Phase 16
C. Onset Of Synchronization 16
D. Contribution From Hydrodynamic Interaction 17
E. Contribution From Optical Interaction 18
F. Notes On Synchronization Mechanism 20

VI. System Sensitivity And Connection With Experiment 20

VII. Comparison With Low Reynolds Numeber Synchronization And The Kuramoto Model 23
A. Kuramoto Model 23
B. Hydrodynamic Synchronization In The Low Reynolds Number Regime 23
C. Synchronization Of Spin Driven Oscillators In Vacuum 24

VIII. Cooling Towards The Quantum Regime 25

IX. Supplementary References 26

References 26

I. PHYSICAL SYSTEM

The physical system considered in this article consists of a pair of counter-propagating circularly polarized Gaussian beams. Their axes are parallel, and separated in the x direction by a distance

$d \approx 8\mu$m. The wavelength is 1064nm and the nominal beam waist radius is 1μm. Each trap holds a polystyrene microsphere with radius $\approx 0.4\mu$m.

II. MODEL AND CALCULATIONS

In this section, we describe the framework for performing the theoretical calculations and simulations presented below. The stochastic motion of the system is modelled by the Langevin equation with inertia, Eq. (1).

$$\mathbf{F}(\mathbf{r}) + \mathbf{F}^L(t) + \boldsymbol{\Xi}(\mathbf{r})\dot{\mathbf{r}} = m\ddot{\mathbf{r}}, \tag{1a}$$

$$\langle \mathbf{F}^L(t)\rangle = 0, \;\; \langle \mathbf{F}^L(t) \otimes \mathbf{F}^L(t')\rangle = 2k_BT\boldsymbol{\Xi}(\mathbf{r})\delta(t-t'). \tag{1b}$$

The coordinates of the centres of mass of the particles are $\mathbf{r} = (\mathbf{r}_1, \mathbf{r}_2, \dots)$, where $\mathbf{r}_i$ are the coordinates for particle i. The corresponding velocities are $\dot{\mathbf{r}} = (\dot{\mathbf{r}}_1, \dot{\mathbf{r}}_2, \dots)$. The systematic, reactive forces, $\mathbf{F}(\mathbf{r}) = (\mathbf{F}_1(\mathbf{r}), \mathbf{F}_2(\mathbf{r}), \dots)$, are induced by the incident optical field. Dissipative forces are linear in the velocity, $\dot{\mathbf{r}}$, and given by $\boldsymbol{\Xi}(\mathbf{r})\dot{\mathbf{r}}$, where $\boldsymbol{\Xi}(\mathbf{r})$ is the resistance matrix, which includes hydrodynamic (or aerodynamic) coupling. The system is subject to delta-correlated stochastic forces, $\mathbf{F}^L(t)$ with zero mean and covariance proportional to $\boldsymbol{\Xi}(\mathbf{r})$, Eq. (1b).
Optical scattering is computed with the Generalized Lorenz Mie Theory (GLMT), with optical interactions computed iteratively [1]. The counter-propagating Gaussian beams are constructed according to the Richards-Wolf formulation [2]. Optical forces are calculated from the total optical fields using the Maxwell Stress Tensor (MST), which is equivalent to both the Abraham and Minowksi tensors [3] when evaluated in vacuum. For simulations, the resistance matrix, $\boldsymbol{\Xi}(\mathbf{r})$, is calculated using the Rotne-Prager tensor [4], with effective viscosity according to [5]. In analysis we use the Oseen tensor [4] to get qualitatively meaningful results,

$$\dot{\mathbf{r}} = \mathbf{H}\mathbf{F}, \tag{2a}$$

$$H_{ij} = \frac{1}{\xi_0}\mathbf{I}\delta_{ij} + \frac{3}{4\xi_0}\frac{a}{r_{ij}}\Big(\mathbf{I} + \hat{\mathbf{r}_{ij}} \otimes \hat{\mathbf{r}_{ij}}\Big)(1-\delta_{ij}). \tag{2b}$$

Parenthetically, we note the analogous treatment for the low Reynolds number case, where the Oseen tensor captures the required dynamics for hydrodynamically coupled spheres in an external potential [6, 7]. In Eq. (2) $\mathbf{r}_{ij} = \mathbf{r}_i - \mathbf{r}_j$ is the displacement vector between the centres of particle j and i, $r_{ij} = |\mathbf{r}_{ij}|$ is its modulus and $\hat{\mathbf{r}}_{ij}/|\mathbf{r}_{ij}|$ is the corresponding unit vector. The Stokes drag of an independent sphere of radius a moving through a medium with effective viscosity μ is $\xi_0 = 6\pi\mu a$. The resistance matrix, $\boldsymbol{\Xi}(r)$ is approximated by the inverse of $\mathbf{H}$, which can be approximated to lowest order as,

$$\boldsymbol{\Xi}_{ij}(\mathbf{r}) \approx \mathbf{H}_{ij}^{-1} \approx \xi_0\mathbf{I} - \frac{3\xi_0}{4}\frac{a}{r_{ij}}\Big(\mathbf{I} + \hat{\mathbf{r}_{ij}} \otimes \hat{\mathbf{r}_{ij}}\Big)(1-\delta_{ij}). \tag{3}$$

In all cases the effective viscosity, μ, is evaluated from kinetic theory [5].
We note that the optical force, $\mathbf{F}$, is proportional to the optical power, P, i.e.

$$\mathbf{F} = P\mathbf{f}, \tag{4}$$

where $\mathbf{f}$ is the optical force per unit power. In addition, the resistance matrix, $\boldsymbol{\Xi}$ is proportional to the viscosity, μ,

$$\boldsymbol{\Xi} = \mu\mathbf{s}, \tag{5}$$

where $\mathbf{s}$ is the resistance matrix normalized by viscosity, depending only the radii of the spheres comprising the system and their relative positions. We write, $\xi_0 = \mu s_0 = 6\pi\mu a$. For a fixed arrangement of optical beams, and a fixed set of microspheres, it is immediately obvious that the dynamical motion can be tuned by two parameters, the optical power, P, and the viscosity, μ. While P controls the magnitude of the systematic forces, μ determines the size of the fluctuations and the dissipative forces.

In the following, we make use of two kinds of computation. The first involves the evaluation of the stiffness matrix for the system (see below). Equilibrium positions for the spheres are found by applying the stabilized Barzilai-Borwein algorithm [8]. Thereafter the stiffness matrix is calculated by numerically approximating the derivatives of the force around the mechanical equilibrium configuration. The second kind of calculation consists of the direct numerical simulation of the system. This is achieved by numerically integrating the Langevin equation, Eq. (1b), with the algorithm given by [9], and extended to multiple particles [10].

In the following we give a general treatment for linear systems, including hydrodynamic interactions, before specializing to the specific system considered here. This corresponds to *sub-threshold* behaviour, where the spheres do not move too far from their equilibrium configuration. The next sections are concerned with the above threshold behaviour, in which non-conservative forces have destabilized the traps and limit cycles have formed. We present detailed simulations of the system and discus the complex mechanism through which these limit cycles synchronize.

III. GENERAL THEORY FOR LINEAR FORCES

The following section combine theory presented in previous papers [11–13]. A related account is provided by Ng. et. al. [14], although this treatment is focused on the stability of large, overdamped systems and neglects hydrodynamic coupling (which appears to be significant in this regime [13]) and thermal fluctuations.

As mentioned previously, systems such as the one treated here, have a configuration ($\mathbf{r}_0$) in which the optical forces vanish, $\mathbf{f}(\mathbf{r}_0) = 0$ and are, loosely speaking, restoring. More precisely, this equilibrium configuration is the set of coordinates that the spheres would settle into if their motion was overdamped and there were no thermal fluctuations. Note, this is a mechanical equilibrium. As described below, it does not necessarily correspond to a thermodynamic equilibrium. In particular, when the forces are locally non-conservative (as is the case here), the Boltzmann distribution is not satisfied in general. Linearizing the optical forces about this equilibrium configuration results in the generalized Hooke's law, Eq. (6),

$$\mathbf{F} \approx -\mathbf{K}\mathbf{q} \equiv -P\mathbf{k}\mathbf{q}, \tag{6a}$$

$$K_{ij} = -\left.\frac{\partial F_i}{\partial x_j}\right|_{\mathbf{q}=0}, \tag{6b}$$

where $\mathbf{q} = \mathbf{r} - \mathbf{r}_0$ are small displacements relative to $\mathbf{r}_0$ and $-\mathbf{K}\mathbf{q}$ is the local linearisation of the force field, $\mathbf{F}$ with *stiffness matrix* $\mathbf{K} = P\mathbf{k}$, where $\mathbf{k}$ is the stiffness per unit power. Note, in Eq. (6b), F_i denotes the ith entry in the vector, $\mathbf{F}$, and x_j the jth entry in the vector $\mathbf{r}$, and a Cartesian reference frame is assumed. In the following, stiffness matrices or matrix elements written in capital letters refer to the absolute quantities while small letters indicate normalization by power. With the linear approximation for the forces, Eq. (1a) becomes

$$-\mathbf{K}\mathbf{q} - \mathbf{\Xi}(\mathbf{r}_0)\dot{\mathbf{q}} + \mathbf{F}^L = m\ddot{\mathbf{q}} \tag{7}$$

In the frequency domain, Eq. (7) is,

$$\big(\mathbf{K} + i\omega\mathbf{\Xi}(\mathbf{r}_0) - \omega^2 m\big)\hat{\mathbf{q}} \equiv \mathbf{M}\hat{\mathbf{q}} = \hat{\mathbf{F}}^L(\omega) \tag{8}$$

where $\hat{\mathbf{F}}$ and $\hat{\mathbf{q}}$ are the Fourier transforms of $\mathbf{F}$ and $\mathbf{q}$. The normalization of $\hat{\mathbf{F}}$ is

$$\langle \hat{\mathbf{F}}^L(\omega)\rangle = 0, \tag{9a}$$

$$\langle \hat{\mathbf{F}}^L(\omega) \otimes \hat{\mathbf{F}}^L(\omega')\rangle = 2k_B T\mathbf{\Xi}\delta(\omega - \omega'). \tag{9b}$$

The power spectral density (PSD) can be formed directly from Eq. (8) and Eq. (9b),

$$\langle \hat{\mathbf{q}}(\omega) \otimes \hat{\mathbf{q}}^*(\omega)\rangle = 2k_B T\mathbf{N}\mathbf{\Xi}\mathbf{N}^H, \tag{10}$$

where $\mathbf{N} = \mathbf{M}^{-1}$ and ^{H} denotes the complex conjugate. Finally, the time dependent correlation functions are given by the Fourier transform of the PSD, according to the Weiner-Khinchine theorem, i.e.

$$\langle \mathbf{q}(t)\mathbf{q}(t+\tau)\rangle = \frac{1}{2\pi}\int_{-\infty}^{\infty} \langle \hat{\mathbf{q}}(\omega) \otimes \hat{\mathbf{q}}^*(\omega)\rangle e^{-i\omega\tau} d\omega. \tag{11}$$

Although this treatment is exact, it does not provide much insight into the stochastic motion executed the particles. To go further, it is helpful to decompose the motion into discrete modes, and examine their variation with power, P, and viscosity, μ. Doing this raises a further problem: the stiffness, $\mathbf{K}$, and hydrodynamic resistance, $\mathbf{\Xi}(\mathbf{r}_0)$ cannot be simultaneously diagonalized. In the next section, we give a perturbation approach that decouples the system into a discrete set of oscillators, each associated with a particular mode and characteristic frequency. From this decomposition, we obtain approximate and explicit expressions for the time dependent correlation functions.

A. Perturbation Theory And Spectral Decomposition

In the following, we use a perturbation approach to reduce the coupled linear system of equations, Eq. (8), to a discrete set of uncoupled oscillators. The perturbation necessarily consists of hydrodynamic coupling, and can also include optical coupling where appropriate. The underlying assumption is that the perturbation is weak and does not alter the main features of the stochastic motion. The first term describes the significant, qualitative features of the stochastic motion and the second, which we treat as a perturbation, is a quantitative correction. First, we separate Eq. (8) into two parts,

$$\big(\mathbf{K}_0 + i\omega\xi_0\mathbf{I} - \omega^2 m\mathbf{I}\big)\hat{\mathbf{q}} + \big(\mathbf{K}_1 + i\omega\mathbf{\Xi}_1\big)\hat{\mathbf{q}} \equiv \mathbf{M}_0\hat{\mathbf{q}} + \mathbf{M}_1\hat{\mathbf{q}} = \hat{\mathbf{F}}^L(\omega), \tag{12}$$

where $\mathbf{K} = \mathbf{K}_0 + \mathbf{K}_1$ and $\mathbf{\Xi} = \xi_0\mathbf{I} + \mathbf{\Xi}_1$ and $\mathbf{M}_0 = (\mathbf{K}_0 + i\omega\xi_0\mathbf{I} - \omega^2 m\mathbf{I})$ and $\mathbf{M}_1 = (\mathbf{K}_1 + i\omega\mathbf{\Xi}_1)$. This separation is made on the basis of the following principles,

1. The spectrum of $\mathbf{K}_0$ should be similar, and close to, the spectrum of $\mathbf{K}$. For each eigenvalue, Λ^0, of $\mathbf{K}_0$, there must be an eigenvalue, Λ of $\mathbf{K}$ where Λ and Λ_0 are close and are either both real or both complex.

2. The second term on the left of Eq. (12) should not qualitatively modify the PSD or correlation functions, only provide a quantitative correction. This is partially ensured by (1). In addition, this second term should be small compared with the first.

To diagonalize the system, Eq. (12), we perform a spectral decomposition of $\mathbf{K}_0 + \mathbf{M}_1$, treating $\mathbf{M}_1$ as a perturbation. That is, we seek the right (Eq. (13a)) and left (Eq. (13b)) eigenvectors as well as the eigenvalues, Λ_i of the system,

$$(\mathbf{K}_0 + \epsilon\mathbf{M}_1)\mathbf{v}_i = \Lambda_i \mathbf{v}_i, \tag{13a}$$

$$\mathbf{w}^T(\mathbf{K}_1 + \epsilon\mathbf{M}_2) = \Lambda_i \mathbf{w}_i^T, \tag{13b}$$

$$\mathbf{w}_i^T \mathbf{v}_j = \delta_{ij} \tag{13c}$$

using the perturbation,

$$\Lambda_i \approx \Lambda_i^0 + \epsilon\Lambda_i^1 + \ldots, \tag{14a}$$

$$\mathbf{v}_i \approx \mathbf{v}_i^0 + \epsilon\mathbf{v}_i^1 + \ldots, \tag{14b}$$

$$\mathbf{w}_i \approx \mathbf{w}_i^0 + \epsilon\mathbf{w}_i^2 + \ldots. \tag{14c}$$

For the right eigenvectors, the lowest orders are,

$$\mathbf{K}_0\mathbf{v}_i^0 = \Lambda_i^0\mathbf{v}_i^0, \tag{15a}$$

$$\mathbf{K}_0\mathbf{v}_i^1 + \mathbf{M}_1\mathbf{v}_i^0 = \Lambda_i^0\mathbf{v}_i^1 + \Lambda_i^1\mathbf{v}_i^0. \tag{15b}$$

Expanding $\mathbf{v}_i^1$ as, $\mathbf{v}_i^1 = \sum_j \alpha_{ij}\mathbf{v}_j^0$ gives,

$$\alpha_{ij}(\Lambda_j^0 - \Lambda_i^0)\mathbf{v}_j^0 + \mathbf{M}_1\mathbf{v}_i^0 = \Lambda_i^1\mathbf{v}_i^0. \tag{16}$$

Taking the scalar product with $\mathbf{w}_k^0$, $k \neq i$ gives,

$$\alpha_{ij} = \frac{(\mathbf{w}_j^0)^T\mathbf{M}_1\mathbf{v}_i^0}{(\Lambda_j^0 - \Lambda_i^0)}, \quad i \neq j \tag{17}$$

and, when $k = i$,

$$\Lambda_i^1 = (\mathbf{w}_i^0)^T\mathbf{M}_1\mathbf{v}_i^0. \tag{18}$$

Expanding the left eigenvectors as $\mathbf{w}_i^1 = \sum_j \beta_{ij}\mathbf{w}_j^0$ and performing similar operations reproduces the expression for Λ_i^1, above and gives,

$$\beta_{ij} = \frac{(\mathbf{w}_i^0)^T\mathbf{M}_1\mathbf{v}_j^0}{(\Lambda_i^0 - \Lambda_j^0)}, \quad i \neq j. \tag{19}$$

Finally, the orthonormality condition for the eigenvectors, Eq. (13c), becomes,

$$\mathbf{w}_n^T\mathbf{v}_m = \delta_{nm} + (\alpha_{mn} + \beta_{nm}), \tag{20}$$

requiring $\alpha_{nn} = \beta_{nn} = 0$. We note that the orthonormality is exact when M_1 is symmetric, since $\alpha_{nm} = -\beta_{mn}$, but gives a small error otherwise. For the current purposes, we are primarily concerned with the corrected eigenvalue, $\Lambda_i \approx \Lambda_i^0 + \epsilon\Lambda_i^1$, which does not suffer this problem. The exact form of the eigenvectors is largely irrelevant, and the eigenvectors of $\mathbf{K}_0$ (i.e. $\mathbf{v}_i^0$ and $\mathbf{w}_i^0$) are sufficient to describe the observations. In addition, we note that the first order perturbation of the specific system studied here (spin driven oscillators) complies with orthonormality as a special case.

Combining the above results, and including the power dependence of the stiffness (i.e. $\mathbf{K} = P\mathbf{k}$) allows us to decompose the total system into a discrete set of one dimensional oscillators,

$$(P\lambda_i + i\omega\mu s_i - \omega^2 m)A_i = \hat{F}_i^L(\omega), \tag{21}$$

where A_i is the frequency domain amplitude of mode i and,

$$\lambda_i \approx \lambda_i^0 + (\mathbf{w}_i^0)^T \mathbf{k}_1 \mathbf{v}_i^0, \tag{22a}$$

$$s_i \approx s_0 + (\mathbf{w}_i^0)^T \mathbf{s}_1 \mathbf{v}_i^0, \tag{22b}$$

where $\mathbf{s}_1$ is the geometric part of $\mathbf{\Xi}_1$, i.e. $\mathbf{\Xi}_1 = \mu\mathbf{s}_1$. Applying a similar perturbation approach to the normalization of the noise in the transformed coordinates gives,

$$\langle \hat{F}_i^L(\omega) \rangle = 0, \tag{23a}$$

$$\langle \hat{F}_i^L(\omega)\hat{F}_i^L(\omega') \rangle \approx 2k_B T \mu s_i \delta(\omega - \omega') \tag{23b}$$

i.e. the noise of each oscillator has a variance proportional to the effective drag. In this picture, each oscillator is effectively independent and one oscillator is not correlated with another. Note: this is a significant assumption. The independence of the oscillators is an artefact of the perturbation approach, which is approximately valid only when $\mathbf{M}_1$ can be treated as a perturbation.
Each oscillator, Eq. (21), has characteristic frequencies given by the roots of the polynomial on the left hand side,

$$\omega_{i\pm} = i\frac{\xi_i}{2m} \pm \frac{1}{2m}\sqrt{4mP\lambda_i - \mu^2 s_i^2}, \tag{24}$$

So the amplitude of the oscillator, Eq. (21), is equivalent to,

$$A_i(\omega) = \frac{F_i^L(\omega)}{m(\omega - \omega_{i+})(\omega - \omega_{i-})}, \tag{25}$$

and the power spectral density is,

$$\langle A_i(\omega)A_i^*(\omega) \rangle = \frac{\langle F_i^L(\omega)F_i^L(\omega') \rangle}{m^2(\omega - \omega_{i+})(\omega - \omega_{i-})(\omega - \omega_{i+}^*)(\omega - \omega_{i-}^*)}. \tag{26}$$

The time dependent correlation functions can then be obtained, via the Wiener-Khinchine theorem, as the Fourier transform of the power spectrum e.g.

$$\langle a_i(t)a_i(t+\tau) \rangle = \frac{1}{2\pi}\int_{-\infty}^{\infty} \langle A_i(\omega)A_i^*(\omega) \rangle e^{-i\omega\tau} d\omega, \tag{27}$$

and the necessary integration evaluated with the residue theorem, using a hemi-circular contour, closed in the lower half plane for $\tau > 0$,

$$\langle a_i(t)a_i(t+\tau) \rangle = \frac{1}{2\pi}\oint \langle A_i(\omega)A_i^*(\omega) \rangle e^{-i\omega\tau} d\omega = i\sum_j \mathrm{Res}(\langle A_i(\omega)A_i^*(\omega) \rangle e^{-i\omega\tau}, \omega_j), \tag{28}$$

where a_i is the time domain modal amplitude. The final form of the PSD (Eq. (26)) and the autocorrelation correlation function (Eq. (27)) depend qualitatively on whether or not the force field is linearly-conservative. The two cases (linearly conservative forces and linearly non-conservative forces) are considered immediately below, before specializing to the physical system considered in this article.

B. Linearly Conservative And Linearly Non-Conservative Systems

As described below, the stochastic motion depends qualitatively on the nature of eigenvalues of the stiffness, $\mathbf{K}$. There are two possibilities:

1. For conservative systems we have $\nabla \times \mathbf{F} = 0$. With $\mathbf{F} = \mathbf{K}\mathbf{q}$, this implies that $\mathbf{K}$ is symmetric i.e. $K_{ij} = K_{ji}$, consistent with the idea that the force is given by the derivative of a potential e.g. $K_{ij} = \partial^2 V(\mathbf{q})/\partial q_j \partial q_i = K_{ji}$. In this case, all the eigenvalues of $\mathbf{K} = P\mathbf{k}$ are real, and the left and right eigenvectors are the same and orthonormal with $(\mathbf{v}_i)^T \mathbf{v}_j = \delta_{ij}$. An eigenvector with a real eigenvalue and identical left and right eigenvectors is a *normal mode*.

2. For linearly non-conservative systems $K_{ij} \neq K_{ji}$ and the left and right eigenvectors are different, in general. Eigenvalues may be real, or they may appear in complex conjugate pairs. An eigenvector with differing left and right eigenvectors is a *quasi-mode* (QM). If its eigenvalue is one of a complex conjugate pair it is a *complex quasi-mode* (CQM).

A general stiffness matrix can be block decomposed into a symmetric part (with real eigenvalues and equal left and right eigenvectors) and a non-symmetric part (with a combination of real and complex eigenvalues and different left and right eigenvectors.

C. Normal Modes

For conservative systems, the eigenvalues, λ_i of $\mathbf{k}$ are necessarily real. As a result, the characteristic frequencies, Eq. (24), satisfy $\omega_{i+} = -\omega_{i-}^*$, and the PSD, Eq. (26), simplifies to the usual Lorentzian,

$$\langle A_i(\omega) A_i^*(\omega) \rangle = \frac{2k_B T \xi_i}{m^2(\omega^2 - \omega_{i+}^2)(\omega^2 - \omega_{i-}^2)}. \tag{29}$$

The autocorrelation function, Eq. (28), becomes [11, 12],

$$\langle a_i(t) a_i(t+\tau) \rangle \approx \frac{k_B T}{P\lambda} \cos\left(\sqrt{P\lambda/m}\tau\right) e^{-\xi\tau/2m}. \tag{30}$$

Several characteristics of these expressions should be noted, which should be compared with the linearly non-conservative case discussed in the following section.

1. For $\tau = 0$, the autocorrelation function, Eq. (27), gives the instantaneous variance and is an expression of the equipartition theorem i.e. the thermal and elastic energies are equal, $k_B T/2 = P\lambda \langle a_i^2 \rangle /2$ i.e. each normal mode oscillator has energy $k_B T/2$, irrespective of optical power or viscosity.

2. As the optical power is increased, the instantaneous variance, $\langle a_i^2 \rangle$ decreases in inverse proportion.

3. The time constant of the exponential decay of the autocorrelation depends on viscosity (through ξ_i), but not on optical power.

D. Quasi-Modes, Complex Eigenvalues

For quasi-modes with real eigenvalues, the PSD and correlation functions are numerically similar to those of normal modes (described above) with the same eigenvalues. Complex eigenvalues induce qualitatively different behaviour. The characteristic frequencies, Eq. (24), for the complex eigenvalue λ have the following form,

$$\omega_{\pm}(\lambda) = i\gamma \pm \Pi = i\big(\gamma \pm \Im(\Pi)\big) \pm \Re(\Pi), \tag{31}$$

where we have suppressed the mode index, i, for brevity, and $\gamma = \xi/2m$ and $\Pi = \sqrt{4mP\lambda - \mu^2 s^2}$. That it, the real parts of the characteristic frequencies the have the same amplitude and the opposite signs, while the imaginary parts, which describe motional damping, have different amplitude but, for high enough viscosity, the same sign. Similarly the characteristic frequencies for the conjugate eigenvalue are,

$$\omega_{\pm}(\lambda^*) = i\big(\gamma \mp \Im(\Pi)\big) \pm \Re(\Pi). \tag{32}$$

When the imaginary part of the characteristic frequency is $\gamma \pm \Im(\lambda)$ the real part is $\pm\Re(\lambda)$ for the eigenvalue λ and $\mp\Re(\lambda)$ for the eigenvalue λ^*. The real part of the characteristic frequency describes oscillation and the imaginary part attenuation. Together, the two quasi-modes, corresponding to the complex conjugate eigenvalues together provide a complete description of the free motion. The characteristic frequencies, $\omega_{i\pm}$ in Eq. (24), have an imaginary part which contains both the viscous drag, ξ_i, and an additive contribution deriving from the imaginary part of the eigenvalue, λ_i. Since the former contribution is proportional to viscosity, and the latter varies with power, the imaginary part of one of the characteristic frequencies can be made equal to zero. From Eq. (24),

$$\begin{aligned}(\omega_{\pm} - i\gamma)^2 &= \pm\Pi, \\ \omega_{\pm}^2 - i\gamma\omega_{\pm} &= \frac{P\lambda}{m}.\end{aligned}$$

Requiring ω_- to be purely real, and comparing real and imaginary parts of the preceding equation gives the following condition for $\Im(\omega_-) = 0$. The same condition applies for the eigenvalue λ^*. For the quasi-mode with index i, the condition for this to occur is,

$$\Im(\omega_{i-}) = 0 \quad \Rightarrow \quad \frac{P_i}{\mu_i^2} = \frac{s^2\Re(\lambda_i)}{m\Im(\lambda_i)^2}. \tag{33}$$

For higher optical powers or lower viscosities, $\Im(\omega_{i-}) < 0$ and the system is unstable i.e.

$$\Im(\omega_{i-}) < 0 \quad \Rightarrow \quad \frac{P_i}{\mu_i^2} > \frac{s^2\Re(\lambda_i)}{m\Im(\lambda_i)^2}. \tag{34}$$

This can be seen qualitatively by taking the under damped approximation, $4m\Re(\lambda_i) \gg \xi_i^2 \equiv \mu^2 s^2$, in Eq. (24),

$$\omega_{i\pm} \approx \pm\sqrt{\frac{P\lambda_i}{m}} + i\frac{\mu s_i}{2m} \tag{35}$$

$$= \pm\sqrt{\frac{P}{m}}\Re(\lambda_i^{1/2}) + i\Big[\frac{\mu s_i}{2m} \pm \sqrt{\frac{P}{m}}\Im(\lambda_i^{1/2})\Big]. \tag{36}$$

The value, P_i/μ^2 in Eq. (33), is a threshold condition. For higher values of P/μ^2 the fixed point of the system becomes unstable. The approach to this threshold is accompanied by a growth in

the peak of the PSD and a reduction of the peak width and half its height [11, 12]. This process dominates the autocorrelation, Eq. (28), so that,

$$\langle a_i(t+\tau)a_i(\tau)\rangle \propto \frac{k_BT}{\Im(\omega_{i-})}e^{i\Re(\omega_{i-})\tau}e^{-\Im(\omega_{i-})\tau}, \tag{37}$$

which increases in amplitude as $\Im(\omega_{i-}) \to 0$. The qualitative features of linearly non-conservative systems are summarized below.

1. The variance, given by Eq. (37) with $\tau = 0$, increases with increasing optical power as the instability is approached and $\Im(\omega_{i-}) \to 0$. In particular, the equipartition theorem is not satisfied and the energy in the quasi-mode increases with optical power.

2. The time constant of the exponential decay of the autocorrelation depends both on viscosity and optical power, and becomes large as the threshold condition, Eq. (33), is approached.

3. As the optical power is increased, for fixed viscosity, the stochastic motion of a system comprising multiple quasi-modes, with differing complex eigenvalues, will become dominated by the quasi-mode with the lowest threshold power, Eq. (33).

These properties should be contrasted with those of conservative systems, listed at the end of the previous section.
Finally, we note a property of quasi-modes with complex conjugate eigenvalues. The left and right eigenvectors associated with λ_i are the complex conjugates of those associated with λ_i^*. These pairs of eigenvectors describe the similar motions, differing only by a phase shift. The physical motion (stochastic or deterministic) is linear super-positions of these two, interconnected, quasi-modes.

IV. SPIN-DRIVEN OSCILLATORS IN THE LINEAR REGIME

In this section we apply the general principles described above to the particular system considered in this article. As described above (Sect. I), the system consists of a pair of parallel, counter-propagating, circularly polarized (CPCP) Gaussian beams. The intensity distribution of each counter-propagating beam consists of a stack of high intensity fringes, created by interference of the counter-propagating beams, each of which lies parallel to the xy plane, oriented normally to the propagation direction, z. The lateral intensity gradients are sufficient to confine micro-spheres within the beams.

A. Stiffness Matrix

We first consider, briefly, a single, isolated CPCP beam containing a single micro-sphere (see [11] for a detailed discussion). The optical forces on the micro-sphere vanishes when its centre lies on the beam axis, either within a high intensity fringe, or between adjacent fringes, dependent on the size of the sphere. Inhomogeneous circular polarization generates azimuthal components of optical momentum [15–18], giving rise to corresponding force components. These non-conservative azimuthal forces couple the x and y motions, leaving the z motion uncoupled, which constitutes a normal mode whose behaviour is described above. Restricting attention to motion in the xy plane, the stiffness is given by the gradient of the force, evaluated at the position at which the force is zero i.e. $\mathbf{K}^{(1)} = -[\nabla\mathbf{f}]^T$,

$$\mathbf{K}^{(1)} = \begin{bmatrix} K_r & K_\phi \\ -K_\phi & K_r \end{bmatrix} \equiv P\mathbf{k}^{(1)} = P\begin{bmatrix} k_r & k_\phi \\ -k_\phi & k_r \end{bmatrix}, \tag{38}$$

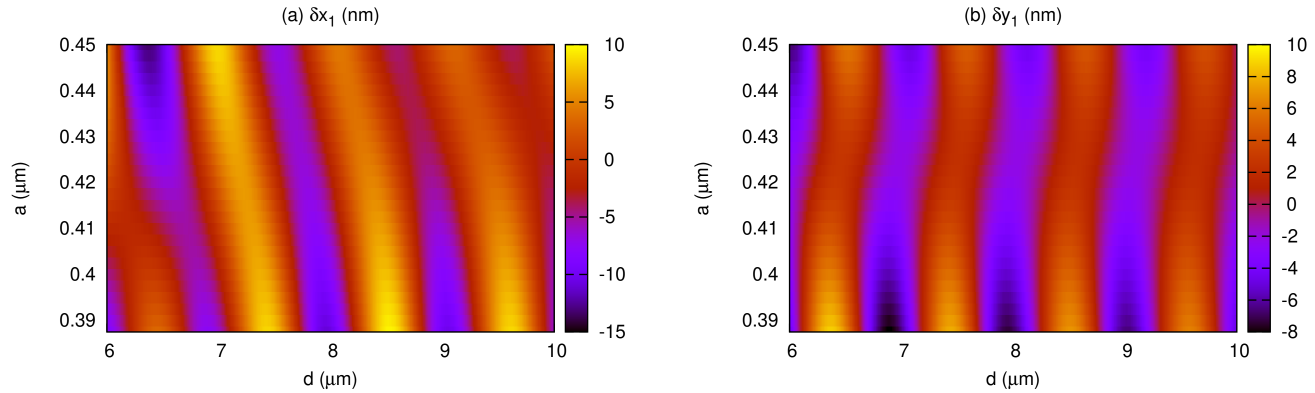

Fig. S1. Mechanical equilibrium positions, δx_1 (a) and δy_1 (b), of a sphere relative to the beam axes, as a function of sphere radius (a) and beam separation (d). Data are plotted for the sphere in the beam whose axis passes through $(-d/2, 0)$. Equilibrium displacements for the second sphere, δx_2 and δy_2 are given by $\delta x_2 = -\delta x_1$ and $\delta y_2 = -\delta y_1$.

where $k_r = -\partial f_x/\partial x = -\partial f_y/\partial y$ is the stiffness for the conservative gradient force and $k_\phi = -\partial f_x/\partial y = \partial f_y/\partial x$ is the stiffness for the azimuthal spin force (ASF).
For a pair of parallel CPCP beams, each containing a single micro-sphere, multiple scattering induces additional forces between the micro-spheres. In the xy plane, the momentum carried by the pair of beams is odd under inversion. This symmetry is reflected both in the mechanical equilibrium configuration (at which the forces vanish), and in the stiffness matrix for the pair. Fig. (S1) shows the small displacements of the spheres in the traps caused by interaction forces: the displacement of the first sphere relative to the beam axis is the negative of that of the second, consistent with the overall system symmetry. The stiffness matrix for the pair, $\mathbf{K}$, has the following form,

$$\mathbf{K} = \begin{bmatrix} \bar{\mathbf{K}}^{(1)} & \mathbf{A} \\ \mathbf{A} & \bar{\mathbf{K}}^{(1)} \end{bmatrix}. \tag{39}$$

Here, $\bar{\mathbf{K}}^{(1)}$ is the stiffness of one of the microspheres with respect to its own displacement. It differs slightly from $\mathbf{K}^{(1)}$, the stiffness in a single CPCP beam, due to interactions with the neighbouring beam and micro-sphere. The sub-matrix $\mathbf{A}$ describes the non-conservative interaction between the micro-spheres. The displacements of the micro-spheres relative to their equilibrium positions are arranged as,

$$\mathbf{q} = (\mathbf{q}_1, \mathbf{q}_2), \tag{40a}$$

$$\mathbf{q}_i = (x_i, y_i) \quad i = 1, 2. \tag{40b}$$

Once again, the power normalized stiffness is written in lower case letters, $\mathbf{K}^{(2)} = P\mathbf{k}^{(2)}$ etc. Calculations of the unique elements of $\mathbf{k}^{(2)}$ are given in Figures (S2) and (S3), for varying beam separation and a range of sphere radii surrounding the default experimental value. The optical interaction imposes a weak, periodic modulation on all stiffness elements. This modulation is strongest for matrix elements connected with displacements in x (the direction of the line connecting the axes of the traps), that is, $k^{(1)}_{xx}$, $k^{(1)}_{yx}$ and a_{xx}, a_{yx}. Elements of $\mathbf{A}$ oscillate about zero for increasing trap separation, d.

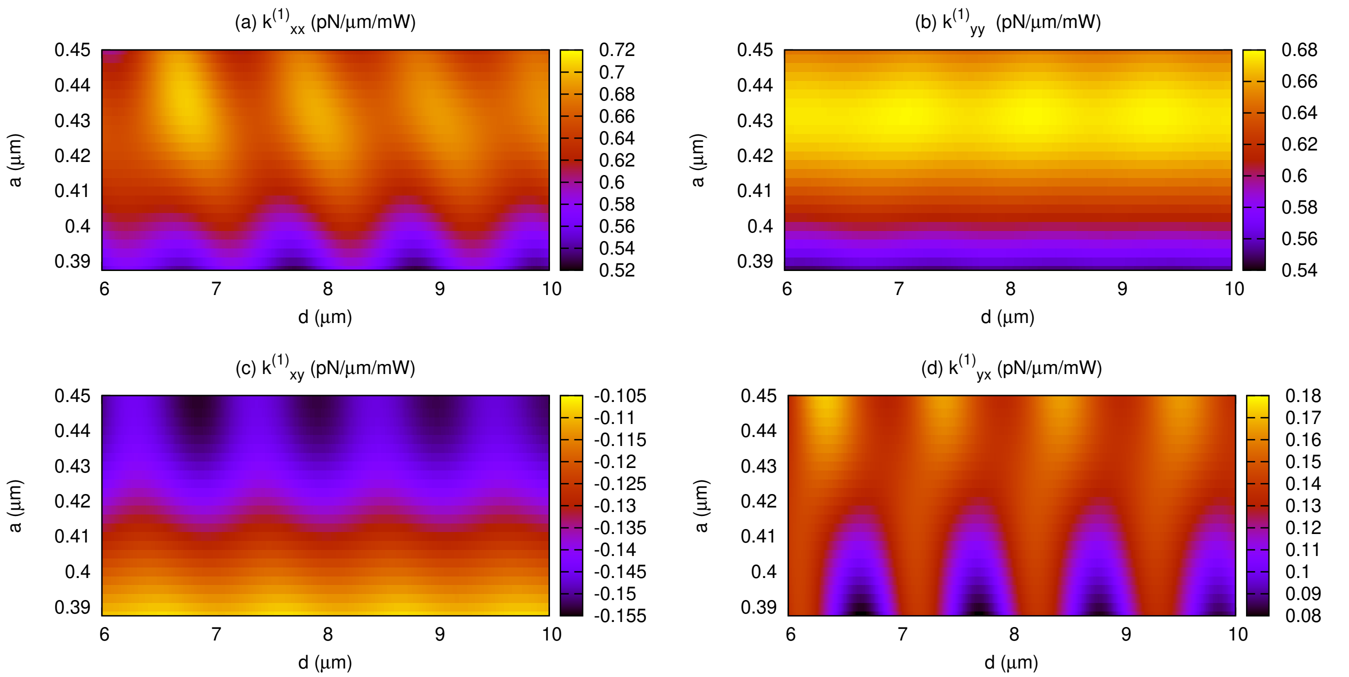

Fig. S2. Elements of the stiffness matrix, $\bar{\mathbf{k}}$, as functions of sphere radius (a) and beam separation (d): $\bar{k}^{(1)}_{xx}$ (a), $\bar{k}^{(1)}_{yy}$ (b), $\bar{k}^{(1)}_{xy}$ (c), $\bar{k}^{(1)}_{yx}$ (d)

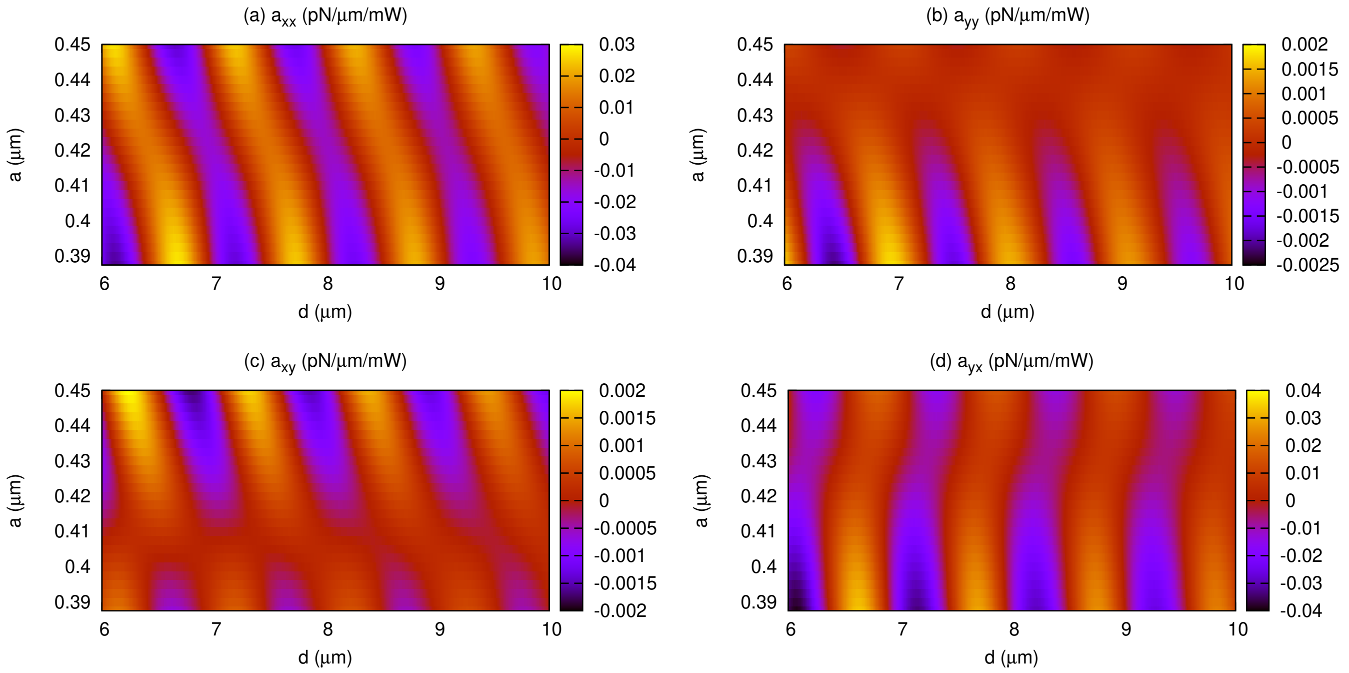

Fig. S3. Elements of the coupling matrix, $\mathbf{a}$, as functions of sphere radius (a) and beam separation (d): a_{xx} (a), a_{yy} (b), a_{xy} (c), a_{yx} (d). The coupling matrix $\mathbf{a}$ is dominated by the elements $a_{xx} = -\partial f^1_x/\partial x_2 = -\partial f^2_x/\partial x_1$ and $a_{yx} = -\partial f^1_y/\partial x_2 = -\partial f^2_y/\partial x_1$ i.e. the x and y components of the force on one sphere, induced by an displacement of the second sphere in the x direction.

B. Hydrodynamic Resistance

We approximate the hydrodynamic resistance using Eq. (3), and take $\mathbf{r}_{ij} \approx d\hat{\mathbf{x}}$, with d the separation between the traps. Then,

$$\boldsymbol{\Xi} = \begin{bmatrix} \boldsymbol{\Xi}_0 & \boldsymbol{\Xi}_X \\ \boldsymbol{\Xi}_X & \boldsymbol{\Xi}_0 \end{bmatrix} \tag{41}$$

with

$$\boldsymbol{\Xi}_0 = \begin{bmatrix} \xi_0 & 0 \\ 0 & \xi_0 \end{bmatrix}, \qquad \boldsymbol{\Xi}_X = \begin{bmatrix} 2\xi_X & 0 \\ 0 & \xi_X \end{bmatrix} \tag{42}$$

with,

$$\xi_0 = 6\pi\mu a, \tag{43a}$$

$$\xi_X = -\frac{3}{4}\frac{a}{d}\xi_0. \tag{43b}$$

C. Stochastic Motion

The PSD and correlation functions can be numerically evaluated, directly from the stiffness matrix (Eq. (39)) and hydrodynamic resistance (Eq. (41)), using the results of Eqns. (26) and (11). However, this does not provide much insight. To go further, note that the form of the stiffness and resistance matrices allow us to decouple the system by transforming to a basis consisting of the *centre of mass* (CoM) and *breathing* (BR) coordinates,

$$\mathbf{q}_c = (\mathbf{q}_1 + \mathbf{q}_2)/\sqrt{2}, \tag{44a}$$

$$\mathbf{q}_b = (\mathbf{q}_1 - \mathbf{q}_2)/\sqrt{2}. \tag{44b}$$

These are analogous to the breathing and centre of mass modes appearing in binary, conservative systems. Here, they refer to intrinsically two dimensional motions, corresponding to the coupled quasi-modes described in Section (III D). The CoM and BR coordinates, or oscillators, each support two QMs. In the following we refer to them as the centre of mass, CoM and the BR quasi-modes. Each pair of QMs are interconnected and, together, they provide a complete description of the stochastic motion in the linear regime. In the new basis,

$$\left[(\bar{\mathbf{K}}^{(1)} \pm \mathbf{A}) + i\omega(\boldsymbol{\Xi}_0 \pm \boldsymbol{\Xi}_X) - \omega^2 m\mathbf{I}\right]\hat{\mathbf{q}}_{c/b} = \hat{\mathbf{F}}^L_{c/b}, \tag{45}$$

where $\mathbf{I}$ is the identity matrix in two dimensions and,

$$\langle\hat{\mathbf{F}}_{c/b}(\omega) \otimes \hat{\mathbf{F}}_{c/b}(\omega') = 2k_BT(\boldsymbol{\Xi}_0 \pm \boldsymbol{\Xi}_X)\delta(\omega - \omega'). \tag{46}$$

An appropriate separation for the stiffness and resistance matrices, following the considerations outlined above (Section (III A)), is,

$$\left[\mathbf{K}^{(1)} + i\omega\xi_0 - \omega^2 m\mathbf{I}\right]\hat{\mathbf{q}}_{c/b} + \left[(\delta\mathbf{K}^{(1)} \pm \mathbf{A}) \pm i\omega\boldsymbol{\Xi}_X\right]\hat{\mathbf{q}}_{c/b} = \hat{\mathbf{F}}^L_{c/b}, \tag{47}$$

where $\delta\mathbf{K}$ is the modification of the stiffness of each trap caused by the interaction with the neighbouring trap i.e. $\bar{\mathbf{K}}^{(1)} = \mathbf{K}^{(1)} + \delta\mathbf{K}^{(1)}$ As before, the first term captures the qualitative behaviour of the system, and the second is a higher order correction which we treat as a perturbation.
Eq. (47) can be interpreted as follows,

1. Neglecting interactions, the first term in Eq. (47)) shows that the CoM and breathing motions behave like a single, isolated spin oscillator, as described previously. In summary, the eigenvalues are complex conjugate pairs, so the system contains an instability. As this instability is approached, stochastic rotations of the particle about the axis grow in amplitude.

2. The second term in Eq. (47), quantifies interactions between the particles. This splits the eigenvalues for the effective stiffness and induces differences in the effective drag for the CoM and breathing quasi-modes, modifying the threshold conditions, Eq. (33). As described in Section (III A), as the optical power is increased, either the amplitude CoM, or the breathing quasi-mode, will grow to dominate.

We discuss these points at greater length below.

1. *Single Spin Oscillators*

Since the oscillators corresponding to the CoM and BR QMs behave similarly to a single, isolated spin-oscillator we first discuss this simpler case. This system has been described previously [11]. The treatment below is consistent with the general framework provided in this paper. To lowest order, the eigenvalues, $\mathbf{K}^{(1)}$, are,

$$\Lambda_{\pm}^{0} = K_r \pm iK_{\phi} \equiv P(k_r \pm ik_{\phi}) \equiv P\lambda_{\pm}^{0} \tag{48}$$

The right eigenvectors are,

$$\mathbf{v}_{\pm}^{0} = (1 \pm i)/\sqrt{2}, \tag{49}$$

and the corresponding left eigenvectors are,

$$\mathbf{w}_{\pm}^{0} = (1 \mp i)/\sqrt{2}. \tag{50}$$

The threshold condition, Eq. (33), is explicitly,

$$\frac{P_X}{\mu_X^2} = \frac{s^2 K_r}{mK_{\phi}^2}, \tag{51}$$

where we define $P_X(\mu_X)$ to be the threshold power and viscosity of a single, isolated spin-oscillator. The autocorrelation function has the form given by Eq. (37), where the dominant motion corresponds to the characteristic frequency for which $\Im(\omega) \to 0$ as the threshold condition is approached. Using Eq. (49), we can transform from the quasi-mode back to Cartesian displacements. In the frequency domain,

$$\langle A_{\pm}A_{\pm}^{*}\rangle = \langle (X_i \pm iY_i)(X_i^{*} \mp iY_i^{*})\rangle = \langle XX^{*}\rangle + \langle YY^{*}\rangle \mp i\langle XY^{*}\rangle \pm i\langle YX^{*}\rangle. \tag{52}$$

Taking the Fourier transform, we see that the imaginary part of the autocorrelation, $\langle a_{\pm}(t+\tau)a_{\pm}(\tau)\rangle$, gives the differential cross covariance, and measures stochastic rotation [19] i.e.

$$\Im\big(\langle a_{+}(t+\tau)a_{+}(t)\rangle\big) = -\Im\big(\langle a_{-}(t+\tau)a_{-}(t)\rangle\big) = \langle x(t+\tau)y(\tau)\rangle - \langle y(t+\tau)x(\tau)\rangle. \tag{53}$$

2. Coupled Spin-driven Oscillators

As described above, the coupled pair can be reduced to two independent spin oscillators, one describing the centre of mass motion, the other describing the breathing motion. Each of these spin oscillators (CoM and breathing) behaves similarly to the single spin oscillator described above. Interactions between the particles split the eigenvalues of the effective stiffness, and the hydrodynamic interactions. Since these quantities determine the threshold condition (Eq. (33)), the CoM and breathing oscillators have differing threshold conditions. For fixed viscosity, the oscillator with the lowest threshold power dominates as the optical power is increased. Using the perturbation expansion (Section (III A)), the corrected eigenvalues are,

$$\lambda_{c+} = \lambda^0_+ + \gamma + \delta, \tag{54}$$

$$\lambda_{c-} = \lambda^0_- + \gamma^* + \delta^*, \tag{55}$$

for the CoM oscillator and,

$$\lambda_{b+} = \lambda^0_+ + \gamma - \delta, \tag{56}$$

$$\lambda_{b-} = \lambda^0_- + \gamma^* - \delta^*, \tag{57}$$

for the breathing oscillator, where,

$$\gamma = \frac{1}{2}\Big[(\delta K^{(1)}_{xx} + \delta K^{(1)}_{yy}) + i(\delta K^{(1)}_{xy} - \delta K^{(1)}_{yx})\Big], \tag{58a}$$

$$\delta = \frac{1}{2}\Big[(A_{xx} + A_{yy}) + i(A_{xy} - A_{yx})\Big]. \tag{58b}$$

The effective hydrodynamic resistances are,

$$\xi_{c/b} = \xi_0\Big(1 \mp \frac{9}{8}\frac{a}{d}\Big) \tag{59}$$

so that the resistance is reduced for the CoM motion, for which the spheres have the same velocity at any point in time, and greater for the breathing motion, for which their velocities are opposite. Using Eq. (33) with the corrected eigenvalues, we can approximate the difference in the threshold powers for fixed viscosity. To lowest order,

$$P_c - P_b \approx -\frac{\xi_0^2}{m}\Big(\frac{4(K_r+\gamma_r)}{(K_\phi+\gamma_i)^3}\delta_i + \frac{9}{2}\frac{a}{d}\frac{(K_r+\gamma_r)}{(K_\phi+\gamma_r)^2}\Big) \tag{60}$$

$$\approx -\frac{\xi_0^2}{m}\Big(\frac{4K_r}{K_\phi^3}\delta_i + \frac{9}{2}\frac{a}{d}\frac{K_r}{K_\phi^2}\Big), \tag{61}$$

where the subscript r (i) denotes the real (imaginary) part of a complex number e.g. $\gamma_i = \Im(\gamma)$. The first term in Eq. (60) relates to the splitting in the eigenvalues between the centre of mass and breathing motion oscillators, and is proportional to $\delta_i = \Im(\delta)$. As can be anticipated from Figs. (S3), δ_i, given by Eq. (58b), oscillates with separation. The second term is derives from the differing hydrodynamic resistances for the breathing and centre of mass motions, and decreases in magnitude monotonically with increasing separation, d. We note that the viscosity μ scales this relationship (through $\xi_0 = 6\pi\mu a$), but does not alter its shape or the locations of the zeroes. When $P_c - P_b < 0$, the centre of mass motion has the lower threshold power and becomes dominant with increasing power. In this way, the effective hydrodynamic resistance favours CoM motion, since it decreases $P_c - P_b$. Fig. (S4) shows a map of $P_c - P_b$ as a function of sphere radius, a and separation, d.

Finally, we note the physical meaning of the dominance of one oscillator (CoM or breathing) over the other. In general, the spheres stochastically rotate about the axis of the beam in which they are confined. When CoM motion dominates, the stochastic rotations are in phase. When the breathing motion dominates, they are in anti-phase.

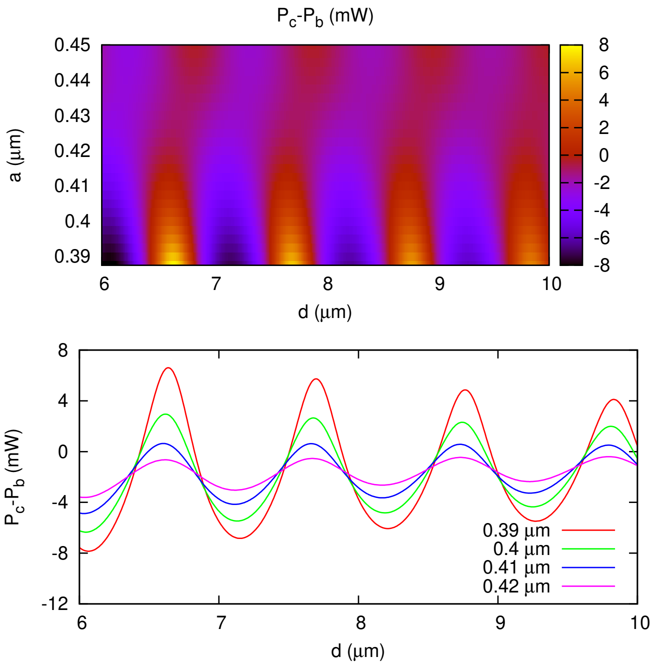

Fig. S4. Plots of $P_c - P_b$ as functions of sphere radius, a, and trap separation, d. *Top Panel:* Heat map of the data, *Lower Panel:* Curves for spheres of four distinct radii, $a = 0.39, 0.4, 0.41$ and 0.42μm.

V. ABOVE THRESHOLD BEHAVIOUR

A. Limit Cycle Formation

As the optical power is increased, the variance of amplitude of the quasi-mode with the lowest (and closest) threshold power grows fastest, and starts to dominate the stochastic motion, see Section (III D). When P exceeds P_i, the fixed point destabilizes and each particle begins to execute noisy, orbits or limit cycles [11, 13], the frequency of which (Ω_O) is given approximately by the real part of the characteristic frequency, $\Re(\omega_i)$, at transition [11, 13], i.e.

$$\Omega_O \approx \sqrt{K_r/m} = \sqrt{Pk_r/m} \tag{62}$$

In the absence of noise, these limit cycles consist of closed curves in a four dimensional phase space, consisting of the x and y coordinates and their respective velocities. Considering a single oscillator, and balancing forces in radial and azimuthal directions gives [11],

$$Pf_\phi = \xi_0 r_O \Omega_O, \tag{63a}$$

$$Pf_r = m r_O \Omega_O^2, \tag{63b}$$

where $f_{r,\phi}$ are the optical forces in radial and azimuthal directions, normalized by the optical power, P, and r_O is the orbit radius. Since Ω_O is approximately independent of power, [11], r_0 is approximately proportional to the power, P, so that the radius of the orbit increases linearly with power, above threshold. Alternatively, we can eliminating Ω_O, top give,

$$r_O = \frac{Pf_\phi^2(r_O)}{\xi_0^2 f_r^2(r_O)}, \tag{64}$$

so that the orbit radius, r_O, increases approximately in proportion with P, as confirmed in [11]. When the orbit radius exceeds the beam waist radius, the limit cycle destabilizes and quasi periodic orbits are formed soon after which the particle is lost from the trap.

In the following we show results of dynamic simulations of interacting particles executing limit cycle oscillations. As discussed above, the simulations are performed with Generalized Lorentz Mie theory [1], combined with stochastic dynamics [9, 10]. These simulations permit us to look at the synchronization mechanism in a little more detail, isolating optical and hydrodynamic contributions.

B. Definition Of Phase

First, we make a note on the definition of the phase of our oscillators. The formal process of phase reduction is well established [20, 21]. The procedure assigns a unique scalar phase to each point on a deterministic limit cycle. It also assigns a unique phase to points on neighbouring trajectories, or *isochrones*, such that these phases are consistent with the phases on the limit cycle. In required, a simple transformation can be applied to the phase to ensure that its rate of change is constant [21]. For our purposes, we adopt the far simpler approach of defining the phase as the azimuthal coordinate of the orbiting particle, relative to a Cartesian frame whose z axis is parallel to the relevant beam axis. We note that our approach lacks the formal rigour of a strict phase reduction and, in effect, ignores deviations from the limit cycle, and neglects velocity (a formal definition of the phase of this oscillator would necessarily include velocity). However, it is intuitive and descriptive, and avoids the complexity that results from analysing the isochrones of the spin oscillators. We note that small deviations from these limit cycles were analysed previously [11]. Extensions of this analysis yield the required formal phase reduction.

C. Onset Of Synchronization

As the optical power is increased, limit cycles form and the particles synchronize. As discussed above, we define the phase simply as the azimuthal coordinate of the particle. We measure the synchronization strength in terms of the relative Shannon entropy [22], S_r. Figure (S5) shows the S_r plotted as a function of the relative power P/P_X for three different viscosities, $\mu_1 = 6 \times 10^{-6}$Pa s, $\mu_2 = 3 \times 10^{-6}$Pa s, and $\mu_3 = 1 \times 10^{-6}$Pa s, where μ_3 corresponds to the experimental value. Two different trap separations are used, $d_1 = 8.22\mu$m and $d_2 = 8.75\mu$m. Higher values of viscosity favour strong synchronization. There are two reasons for this. First, the threshold optical powers, and the optical power required to sustain an orbit of a given radius are higher for higher viscosity, so the optical interaction forces are greater. Second, the hydrodynamic coupling is stronger. As described below, both of these forms of interaction contribute to the synchronization mechanism. The curves in Fig. (S5) come from simulations over time periods of 0.4s. In each case, we note the data points appear to jump between two sets of values. This is due to the stochastic and finite nature of the simulations. The simulation finds its way, randomly, into one of two states with differing synchronization states. Fig. (S6) shows sets of probability distributions of the relative phase, $P(\Delta\phi)d\Delta\phi$, obtained from 0.4s simulations with viscosity μ_3, and trap separations d_1 and d_2. The state for which the probability distribution is peaked at zero is the corresponds to the strongly synchronized state with $S_r \approx 0.3$, while the second, phase locked state has $S_r \approx 0.1$. Overall, it appears that the system randomly accesses a two different states, and that the synchronization strength, S_r, of each of these state increases abruptly as the power crosses a threshold value, P_X. Thereafter S_r increases gradually with increasing power.

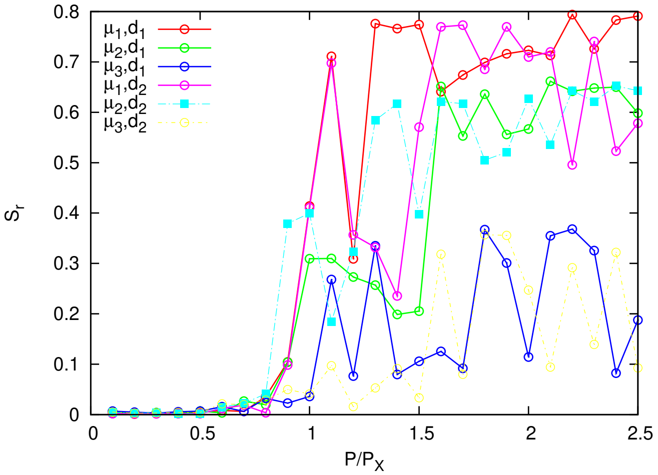

Fig. S5. Plots of synchronization strength against relative power, P/P_X, for two different trap separations, $d_1 = 8.22\mu$m and $d_2 = 8.75\mu$m, and viscosities $\mu_1 = 6 \times 10^{-6}$Pa s, $\mu_2 = 3 \times 10^{-6}$ and $\mu_3 = 1 \times 10^{-6}$.

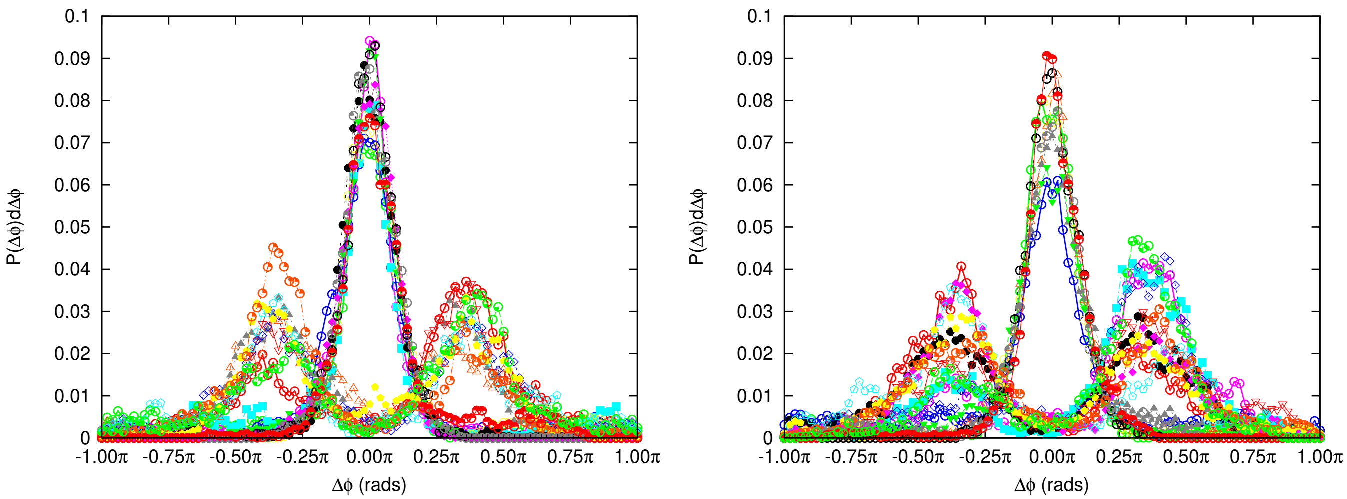

Fig. S6. Probability distribution functions, $P(\Delta\phi)d\Delta(\phi)$, extracted from multiple stochastic simulations. In each case a viscosity of $\mu = \mu_3 = 1 \times 10^{-6}$Pa s is used, and a value of $P/P_X = 1.5$ assumed. *Left Hand Side:* $d = d_1 = 8.22\mu$m. *Right Hand Side:* $d = d_2 = 8.75\mu$m

D. Contribution From Hydrodynamic Interaction

Turning off the optical interaction, we find that the system is still able to synchronize. Figure (S7) shows the relative Shannon entropy, S_r, as a function of P/P_X for varying viscosities. The left hand panel shows results for perfect, counter-propagating Gaussian beams. On the right are results for which the azimuthal optical force, f_ϕ, has been reduced by a factor of four relative to the radial force, so as to reproduce experimental values for P_X. The simulations include only the hydrodynamic interaction, the optical interaction being switched off. In each case, the synchronized state corresponds to $\Delta\phi \approx 0$. This is a surprising result since it is well established that hydrodynamic interactions cannot cannot synchronize rotors moving on fixed circular paths, in the over damped regime [23, 24]. In addition, viscous forces act on velocities not on positions. The

underlying mechanism involves the complex interaction between the shape and size of the limit cycle, the drag on each particle and the inertia. Although the effect warrants further study it appears that the system attempts to minimize the rate of dissipation, in analogy to the minimun dissipation theorem of Onsager [25], or a generalization thereof . More generally, the system may be satisfying some extremal condition governing the formation of this non-equilibrium steady state [26].

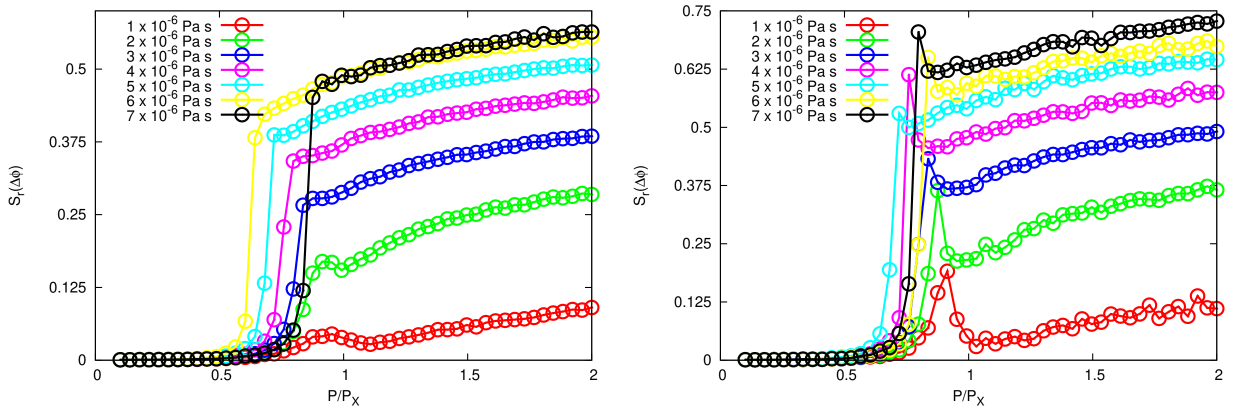

Fig. S7.) Plots of relative Shannon entropy, S_r, against P/P_X for a range of viscosities, μ. Only hydrodynamic interactions alone are included in the simulations. Optical interactions are set to zero. *Left Hand Panel:* Results for ideal counter-propagating Gaussian beams, *Right Hand Panel:* Similar results, with azimuthal forces artificially reduced by a factor of 4 to reproduce experimentally measured values of P_X.

E. Contribution From Optical Interaction

A phenomenological model for our system can be derived in direct analogy with the paradigmatic Kurumato model with inertia and noise [27]. Representing the phase of the limit cycle by the azimuthal coordinate, ϕ, of each microsphere and ignoring variation in the radial coordinate is approximately constant, the equation of motion for the phase of oscillator i is,

$$f_\phi^i + f_\phi^{ij}(\phi_1, \phi_2) - \xi_0 \dot{\phi}_i + \xi_c(\phi_1, \phi_2)\dot{\phi}_j + f_\phi^L(t) = m\ddot{\phi}_i, \tag{65}$$

for $i = 1, 2$ and $j = 3 - i$. Here, f_ϕ^i represents the constant azimuthal force, specific to the radius of the particle orbit, $f_\phi^{ij}(\phi_1, \phi_2)$ is the coupling force acting on i, due to optical interaction with j. Fig. (S8) shows numerically calculated resonant optical coupling forces for deterministic limit cycles formed at $P/P_X = 1.5$, corresponding to orbital radii, $r_O \approx 0.7\mu$m. Two separations, $d_1 = 8.22\mu$m and $d_2 = 8.75\mu$m are used, showing the dependence of the coupling forces on separation. $\xi_0\dot{\phi}_i$ is the Stokes drag on i and $\xi_c(\phi_1, \phi_2)\dot{\phi}_j$ is the hydrodynamic coupling force acting on i due to the motion of j. Subtracting the equation of motion for ϕ_j from that for ϕ_i leads to,

$$f_\phi + f_\phi^c(\Delta\phi) - \xi_0\dot{\phi} + \xi_c(\Delta\phi)\dot{\Delta\phi} + f^L(t) = m\ddot{\Delta\phi}, \tag{66}$$

where $\Delta\phi = \phi_1 - \phi_2$ and the resonant terms have been extracted from the hydrodynamic and optical interactions. These resonant forces correspond to the terms in the Fourier expansion of $f_\phi^{ij}(\phi_1, \phi_2)$ in $\Delta\phi$ [21]. Physically, the resonant force is that part of force that determines the slow evolution of $\Delta\phi$, the remaining terms fluctuating very rapidly and averaging, effectively to zero [21]. Fig. (S9) shows resonant forces for a series of limit cycles formed at a succession of powers, P/P_X,

for which the orbit radius, r_O, increases steadily towards the beam waist radius, $w_0 = 1\mu$m. Curves are plotted for $d = d_1 = 8.22\mu$m (left hand panel) and $d = d_2 = 8.75\mu$m (right hand panel). In the absence of noise, steady state solutions correspond to the condition, $f_\phi + f_\phi^c = 0$ with restoring forces. When each oscillator has the same driving force (i.e. $f_\phi^1 = f_\phi^2$, so that $f_\phi = f_\phi^1 = f_\phi^2 = 0$), steady solutions correspond to the zeroes in the coupling force, $f_\phi^c(\Delta\phi) = 0$ and, when $f_\phi \neq 0$, they correspond to the intersection of the coupling and driving forces. This constitutes synchronization and phase locking. For sufficiently strong driving, the magnitude of f_ϕ falls outside the range of $f^c(\phi)$ and no synchronized states appear. This simplified model corresponds to the well known problem of underdamped Brownian in a tilted periodic potential [28], which is characterized by the following probability distribution,

$$P(\Delta\phi) = \frac{1}{N}\int_{\phi}^{\phi+2\pi} \exp\left(\frac{V(\Delta\phi') - V(\Delta\phi)}{k_B T}\right)(\xi_0 + \xi_c(\Delta\phi))d\Delta\phi', \tag{67}$$

where $V(\Delta\phi)$ is the effective potential, given by the integral of the force, and the effects of inertia have been ignored. We note that $\xi_c(\Delta\phi)$ is a small correction here, and does not qualitatively alter $P(\phi)$. The distribution is sharply peaked at potential minima, where the force, $f_\phi + f_\phi^c(\phi)$ vanishes. Although this is a steady state distribution, it is not at equilibrium and a constant probability flux exists,

$$S = \frac{k_B T}{N}\left(1 - e^{2\pi\Delta V/k_B T}\right), \tag{68}$$

where $\Delta V = V(2\pi) - V(0)$ is the change in the effective potential as ϕ executes one complete cycle. Thus, when the oscillators are perfectly symmetric and the constant forces, f_ϕ^1 and f_ϕ^2 are identical, the flux, S, is zero. When the constant forces are dissimilar, i.e. $f_\phi \neq 0$, the flux is non-zero, and one rotor rotates more rapidly when averaged over time. Physically, the oscillator pair spends most of its time in a synchronized state, occasionally hopping to a neighbouring state, or undergoing a complete 2π phase slip, induced by thermal fluctuations. The process is analogous to Kramers hopping although, in this case, the transitions are made between non-equilibrium states. When the effective driving force, $f_\phi = f_\phi^1 - f_\phi^2 \neq 0$ these transitions occur, on average, in a preferred direction giving rise to the probablility current, S, Eq. (68). When $f_\phi = 0$, hopping may still occur, but there is no preferred direction.

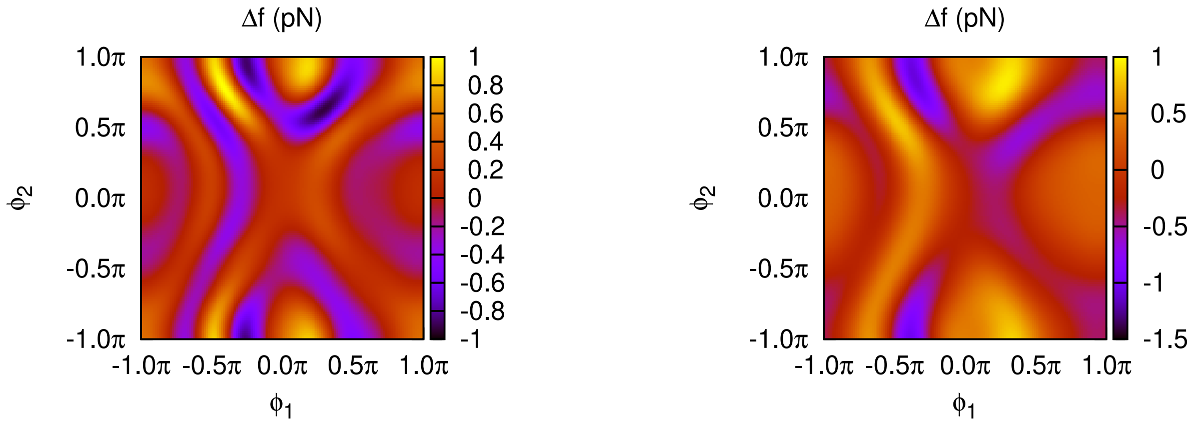

Fig. S8. Optical coupling forces, $f_\phi^{ij}(\phi_1, \phi_2)$per Watt of optical power, $P/P_X = 1.5$ equating to $r_O \approx 0.6\mu$m. *Left Hand Panel:* trap separation, $d = d_1 = 8.22\mu$m. *Right Hand Panel:* trap separation, $d = d_1 = 8.75\mu$m.

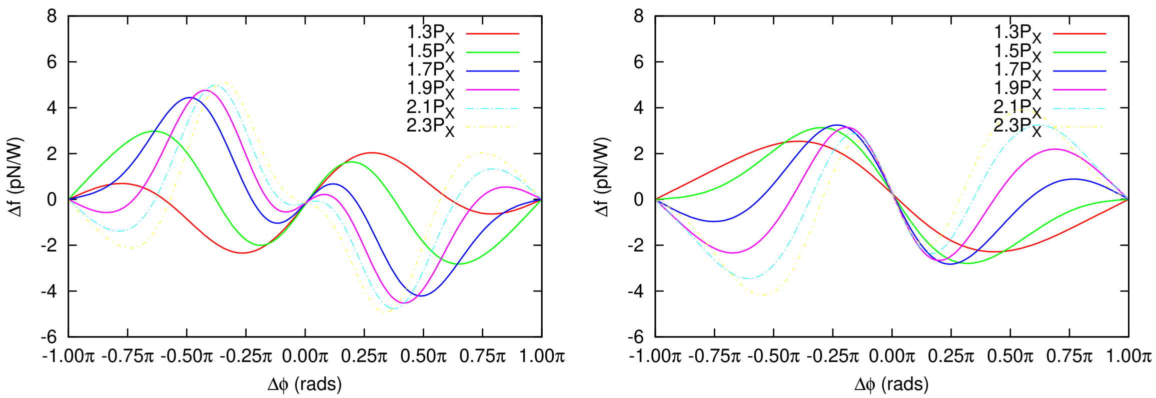

Fig. S9. Resonant force curves, $f^c_\phi(\Delta\phi)$ for increasing P/P_X.*Left Hand Panel:* trap separation, $d = d_1 = 8.22\mu$m. *Right Hand Panel:* trap separation, $d = d_1 = 8.75\mu$m.

We note that this simple approach has some descriptive power. However, the probability distribution functions obtained for this model, either through numerical integration of Eq. (66), evaluation of the approximate expression, Eq. (67), or semi-analytically following [28] do not correlate either with those obtained in the full simulations described above (Section (V C)), or with those observed in the experiment.

F. Notes On Synchronization Mechanism

We make the following comments about the synchronization mechanism operating in this system.

1. In the experiment, synchronization strength increases with optical power. The synchronized state seems to correspond to a unique value of $\Delta\phi$. When phase slips occur they are over intervals of 2π.

2. Simulations also show that synchronization strength increases with optical power.

3. Hydrodynamic interactions alone can cause synchronization in this underdamped regime.

4. Reduction to a simple, one dimensional Kuramoto type model has descriptive power but does not capture experiments or detailed simulations.

Overall, it appears likely that the synchronization observed in our system is driven by hydrodynamic interactions, and subsequently modified by optical interactions. These two mechanisms appear to act cooperatively.

VI. SYSTEM SENSITIVITY AND CONNECTION WITH EXPERIMENT

In the following, we make some comments about the sensitivity of the system to small uncertainties in the defining parameters. First, we review the assumptions made in the model, as well as the measures taken to attempt to correct non-idealities in the experiment. As described in section (II), we make use of an idealised model. The model relies on a number of assumptions, reiterated below, for emphasis. It is asumed that:

1. The circularly polarized optical beams are free from aberrations. That is, the two beams are identical and both are cylindrically symmetric, having a circular cross-section, for which the intensity distribution is Gaussian. In the experiment, we use the spatial light modulator to correct for aberrations [29]. However, small reflections ($\approx 8-10\,\%$) from the vacuum chamber windows unavoidably perturb the beam, generating off-axis aberrations. Note that for perfect, circular beams, the particle trajectories would also be close to circular: the observable deformations in the particle trajectories confirm the existence of aberrations.

2. The particles are identical spheres, with known refractive index, density and radius.

3. The pressure measured at the narrow cuvette accurately measures the pressure within the cell, in the region surrounding the experiment. In addition, the pressure can be used to accurately derive the drag on the particle, via existing relationships [5]. Note: in previous experimental work [10], we have measured the effective drag acting on a sphere of known size. Our results revealed a factor of ≈ 10 difference between experiment and theory. We therefore expect the model to be accurate to within an order of magnitude, but significant deviations may appear.

4. The hydrodynamic coupling can be approximated via the Oseen tensor [4]. We note that previous measurements show significant hydrodynamic coupling [10], with the Oseen tensor providing qualitative insight, but underestimating the magnitude of the effect when compared with measurement.

Despite the steps taken to control the experiment, the system we study here is intrinsically sensitive. The reason for this is that the steady state motion, whether below or above threshold, is derived from a delicate balance between weak non-conservative forces and dissipation: at steady state, the rate of energy transferred to the particle from the non-conservative force must be, on average, equal to the rate at which it is dissipated into the ambient gas. Small changes in either the non-conservative or dissipative forces can shift this balance substantially. This can be most clearly seen in the expression for the threshold conditions (see section (III)),

$$\frac{P_i}{\xi_i^2} = \frac{\Re(\lambda_i)}{m\Im(\lambda_i)}, \tag{69}$$

where i indexes the quasi-mode. Two factors contribute to the sensitivity of the system. First, the threshold power, P_i, depends on the square of the effective drag, ξ_i. As described above, ξ_i is approximately given by the sum of the Stoke's drag on an individual particle, and a second contribution connected with the effective hydrodynamic interaction, Eq. (59), which is proportional to a/d, and about an order of magnitude smaller than the Stoke's drag. The second source of sensitivity is the imaginary part of the eigenvalue, $\Im(\lambda_i)$. As described in section (III), the real part of the eigenvalue, $\Re(\lambda_i)$, is dominated by the gradient force, in combination with a smaller interaction term. The imaginary part is connected with the azimuthal force (which is $\approx 10\%$ of the gradient force), in combination with an interaction term (which is $\approx 1\%$ of the gradient force). The observed dynamics are determined by these small interaction terms which appear in the dissipative forces (i.e. the drag) and the reactive (optical) forces. In both cases, these factors are squared in the threshold condition, so that the observed motion depends delicately on these terms.

As described above, there is some uncertainty in the Stokes drag, and the hydrodynamic interaction. The sensitivity of the optical forces is more complicated. To assess its impact, we consider the threshold condition for a single oscillator with an elliptical cross-section, having an aspect ration ϵ,

$$\epsilon = \frac{w_{0y}}{w_{0x}}, \tag{70}$$

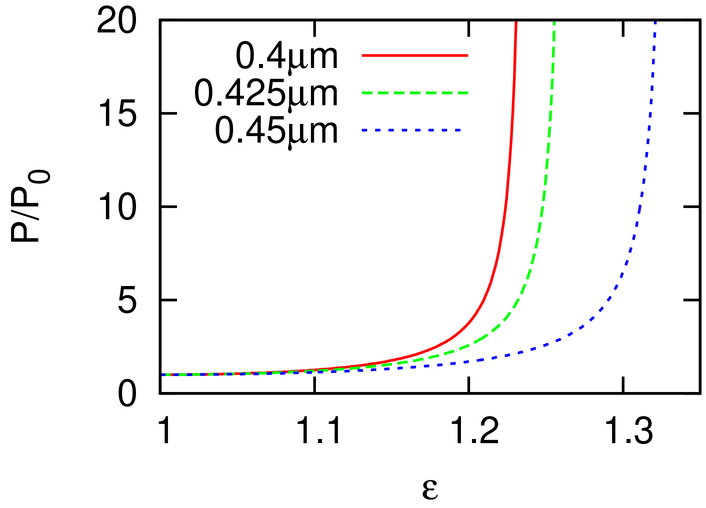

Fig. S10. Threshold power (P) in an elliptical beam, relative to that of a circular beam of the same cross-sectional area (P_0) as a function of the aspect ratio of the elliptic cross section, ϵ.

where w_{0x} and w_{0y} are the beam waist radii in the focal plane, in the x and y directions respectively. The cross-sectional area of the beam is held constant, i.e. $w_{0x}w_{0y}$ is constant. The eigenvalues of the perturbed system are now functions of ϵ. In Figure (S10), we plot the ration of the threshold power, $P(\epsilon)$, for the deformed beam, to that of the circular beam, $P_0 \equiv P(1)$, as a function of ϵ, for three different sphere radii.

The calculations, Fig. (S10), suggest that substantial variation in the threshold power may result from relatively small aberrations in the optical beams. This is compounded by the uncertainty in the drag. Together, these factors may explain the observed discrepency between the theoretical results, for which the threshold power is ≈ 20 mW, and the experiment, for which the threshold power is ≈ 200 mW.

Despite the quantitative difference between the model and experiment, we note that the qualitative agreement is sound. Both the model and experimental systems exhibit a threshold power, below which we observed biased stochastic motion for which the motion of the separate particles is increasingly highly correlated, with one form of correlation dominating the other. Above threshold, the qualitative similarity is maintained, with both systems executing robust, synchronized motion. As a matter of necessity, both the model and experiment attain steady states in which the viscous dissipation is balanced by the gain in kinetic energy from the non-conservative forces. The main difference between experiment and theory lies in the relative influence of hydrodynamic coupling and optical forces. In particular, the former will be lower relative to the latter, in the experiment. For this reason, the CoM mode is strongly favoured strongly in the simulations, since the hydrodynamic interactions are relatively stronger than in the experiment, encouraging minimization of dissipative forces. In contrast, the optical forces tend are relatively stronger in the experiment, so that the synchronization mechanism is more dependent on optical interactions, which give greater weight to synchronized states based on the BR more. Finally, we note that synchronized states based on BR or CoM modes are readily obtained in the experiment, dependent on the beam separation, d, which confirms the active role played by the optical interaction, which naturally oscillates with d.

VII. COMPARISON WITH LOW REYNOLDS NUMEBER SYNCHRONIZATION AND THE KURAMOTO MODEL

The results presented here should be compared with the substantial body of work that exists on the subject of hydrodynamic synchronization in the low Reynolds number regime [30]. The significance of this prior work rests on two principle factors: applications to biological systems, and the nature of the synchronization mechanism itself. In a sense, the system studied in this article is more general than those studied in connection with low Reynolds number synchronization. For example, the dynamics in our system relies on a delicate balance between inertial, reactive (i.e. optical) and dissipative (i.e. viscous) forces, both of which are coupled. In contrast, low Reynolds number hydrodynamic synchonization excludes reactive coupling and inertia. Arguably, it is this specialization which makes the low Reynolds number systems interesting and relevant to biology. The system we report is more general, but lacks the biological applications. Below, we make some remarks about the existing work on low Reynolds number hydrodynamic synchronization, in comparison with the system reported here, and with the paradigmatic Kuramoto model referred to above (section (V E)).

A. Kuramoto Model

As mentioned above (see section (V E)), the Kuramoto model is a paradigmatic model for the synchronization of ensembles of limit cycle oscillators. Noise and inertia may be included [27]. In the latter case, appropriate Fokker-Plank equations can be constructed to explore statistical distributions. Although the equations of motion appear purely symbolic, they can be thought of as general equations deriving from the process of phase reduction, applied to more general and realistic oscillators. Due to the manner in which it is constructed, synchronization in the Kuramoto model is pincipally derived from reactive forces that both drive and couple the oscillators. Synchronization via hydrodynamic coupling is not readily included in the Kuramoto model and naive attempts to do so directly result only in small corrections to the uncoupled probability density functions, Eq. (67).

B. Hydrodynamic Synchronization In The Low Reynolds Number Regime

Synchronization of mesoscopic oscillators in the low Reynolds number regime is of fundamental biological importance due to its connection with swimming bacteria, and fluid transport in, for example, the respiratory systems of animals [30, 31]. The efficiency of both swimming and fluid transport are related to the coordinated i.e. synchronized motions of cilia and flagella. In contrast to the Kuramoto model, discussed above, reactive forces may drive the motions of individual entities, but they do not contribute to coupling. Thus, it was hypotehsised that hydrodynamic forces were responsible for synchronization. However, viscous, dissipative forces are proportional to velocities and, prior to the seminal works described in [30], it was unclear how they could cause the observed coordination of the positions and configurations of small biological entities. Insight into the underlying mechanisms was developed through experimental work using, for example, optical tweezers, in combination with analytical and computational methods. Subsequently, low Reynolds number synchronization has been demonstrated in a range of systems including configuration dependent geomeric switches, rotating helices and rotors, and driven chains of colloidal magnetic beads [32–35]. In essence, synchronization in this regime arises from the dependence of the viscous forces on the system configuration.
Experimental and theoretical studies of minimal models for synchronzing flagella are of particular

relevance here. Such models consist of two (or more) microspheres, driven through a viscous fluid, about periodic trajectories. Experimental work makes use of optical tweezers to provide the required forces. By measuring the positions of the particles, optical traps can be actively repositioned in order to apply prescribed force profiles. Key findings of this work are:

1. Hydrodynamic synchronization is facilitated by flexibility in the prescribed trajectories [36, 37], although it is not essential. Typically, harmonic restoring forces are used to bind particles to a particular path.

2. For circular paths, spatially modulated forces are required [38], and facilitated by flexibility in the periodic paths [36]

3. Synchronization on circular paths depends on the configuration dependence of hydrodynamic coupling. When the phase of one particle lags behind that of another, the paths deform slightly (making use of the flexibility referred to above). In doing so, the leading particle slows a little, and the trailing particle speeds up, restoring synchrony [24].

4. Optimal synchronization is promoted by tuning force modulation and radial flexibility [24, 37].

C. Synchronization Of Spin Driven Oscillators In Vacuum

The system studied in the current article is fundamentally different from that features in either the Kuramoto model, or in the existing work on low Reynolds number synchronization. Nevertheless, the underlying mechanism operating in our system bears some similarity to the mechanisms operating both in the Kuramoto model and in the low Reynolds number case.

a. Comparison with the Kurmaoto model with inertia and noise:

1. As described above, synchronization through hydrodynamic coupling is not readily included in the Kuramoto model, generating only a small correction to the uncoupled probability distribution function, Eq. (67).

2. Our system is intrinsically multidimensional. In the Kuramoto model, reactive forces are a fixed function of the phases of the particles. In our system, perturbations in the trajectory can result in substantial changes to the reactive forces.

b. Comparison with existing work on low Reynolds number systems:

1. Our system operates in the underdamped regime. At steady state, the statisics are determined by a balance between viscous dissipation, inertial forces and non-conservative optical forces.

2. The model systems studied in connection with low Reynolds number synchronization are heavily constrained. In particular, the trajectories followed by the particles, and the modulation of the applied forces are imposed on the systems. In contrast, our system is completely free, autonomous and spontaneous. The trajectories followed by each particle, and the relationship between the trajectories are determined by the equations of motion (including autonomous reactive forces, dissipative hydrodynamic forces and fluctuations) i.e. the system attains a steady state determined soley by physical principles, consistent with stochastic thermodynamics.

Despite these distincions, it appears that the synchronization mechanism operating in our system may include features from both the Kuramoto model and the established mechanisms for hydrodynamic synchronization. For example,

1. In our system, the steady state stochastic dynamics involve a balance between inertial, reactive and dissipative forces, in the presence of fluctuations. The system is free to find optimal paths for the limit cycles and for the relationship between the motion of the particles. Since the hydrodynamic (i.e. dissipative) forces depend on configuration, these optimal trajectories must be influenced by hydrodynamic coupling, to some degree. This process is analogous to the deformations to the paths suffered by particles as synchrony is restored in the low Reynolds number case (see above and [24]). The exact nature of hydrodynamic interactions in this regime is uncertain. Its effect cannot be easily extracted from experimental trajectories in non-conservative systems, since the gain (from non-conservative forces) and loss mechanisms (dissipation) do not vary independently. However, we have measured hydrodynamic interactions in this regime, previously, in a conservative system where their influence can be measured more directly (since there is no mechanism for gain).

2. Reactive forces also play a role in our system. This can be seen by the dependence of the dynamics on the optical power and beam separation. In particular, we see qualitative changes in the synchronization state as we alter the beam separation by small distances on the scale of a wavelength. It is also apparent in the simulations and calculations. As with the Kuramoto model, the ressonant part of the azimuthal component of the reactive force interaction must dominate (see section V E). As noted above, it cannot account entirely for the observed results. A more thorough understanding of the role of reactive forces in our system would need to take into account deformations of the particle trajectories (due, for example, to hydrodynamic interactions), which substantially effect the forces acting on the particles.

VIII. COOLING TOWARDS THE QUANTUM REGIME

The experiments presented in this article take place at pressures for which thermal fluctuations are far too large to sustain quantum effects. To approach the quantum regime would require us to operate at far lower pressures and to implement an appropriate protocol to cool the motion. Routes to achieving these requirements are described below.

1. The scaling behaviour of spin driven oscillators has been described previously [11]. As the particle size is reduced, the magnitude of the azimuthal spin forces, relative to the optical gradient forces decreases towards zero. In particular, for dipolar particles grdient forces are proportional to the volume of the particle, and azimuthal forces are proportional to the square of the volume. Thus, for a fixed optical power, the threshold pressure (or viscosity), necessary for limit cycle formation, can be made arbitrarily small. Switching to nanoparticles presents a number of additional challenges, but would permit synchronization experiments to be performed in ultra high vacuum.

2. In a previous article [39], we show that parametrically modulated optical force fields can effectively cool limit cycles, resulting in extremely coherent, almost deterministic execution of periodic trajectories. Although this effect depends on particular properties of the force

profile, these could be replicated in any system using beam shaping methods.

3. Scattering into a resonant cavity could provide further cooling, as it does in the case of ground state cooling, for example.

Together, these techniques could enable the experimental study of meso-scale synchronization under conditions which approach the quantum regime.

IX. SUPPLEMENTARY REFERENCES

[1] Mishchenko, M. I., Travis, L. D. & Lacis, A. A. *Multiple scattering of light by particles: radiative transfer and coherent backscattering* (Cambridge University Press, 2006).

[2] Richards, B. & Wolf, E. Electromagnetic diffraction in optical systems, ii. structure of the image field in an aplanatic system. *P. Roy. Soc. Lond. A Mat.* **253**, 358–379 (1959).

[3] Brevik, I. Experiments in phenomenological electrodynamics and the electromagnetic energy-momentum tensor. *Phys. Rep.* **52**, 133–201 (1979).

[4] Kim, S. & Karrila, S. J. *Microhydrodynamics: principles and selected applications* (Courier Corporation, 2013).

[5] Beresnev, S., Chernyak, V. & Fomyagin, G. Motion of a spherical particle in a rarefied gas. part 2. drag and thermal polarization. *J. Fluid Mech.* **219**, 405–421 (1990).

[6] Polin, M., Grier, D. G. & Quake, S. R. Anomalous vibrational dispersion in holographically trapped colloidal arrays. *Phys. Rev. Lett.* **96**, 088101 (2006).

[7] Meiners, J.-C. & Quake, S. R. Direct measurement of hydrodynamic cross correlations between two particles in an external potential. *Phys. Rev. Lett.* **82**, 2211 (1999).

[8] Burdakov, O., Dai, Y. & Huang, N. Stabilized barzilai-borwein method. *J. Comput. Math.* **37**, 916–936 (2019).

[9] Grønbech-Jensen, N. & Farago, O. A simple and effective verlet-type algorithm for simulating langevin dynamics. *Mol. Phys.* **111**, 983–991 (2013).

[10] Svak, V. *et al.* Stochastic dynamics of optically bound matter levitated in vacuum. *Optica* **8**, 220–229 (2021).

[11] Svak, V. *et al.* Transverse spin forces and non-equilibrium particle dynamics in a circularly polarized vacuum optical trap. *Nat. Commun.* **9**, 1–8 (2018).

[12] Arita, Y., Simpson, S. H., Zemánek, P. & Dholakia, K. Coherent oscillations of a levitated birefringent microsphere in vacuum driven by nonconservative rotation-translation coupling. *Sci. Adv.* **6**, eaaz9858 (2020).

[13] Simpson, S. H., Arita, Y., Dholakia, K. & Zemánek, P. Stochastic hopf bifurcations in vacuum optical tweezers. *Phys. Rev. A* **104**, 043518 (2021).

[14] Li, X., Liu, Y., Lin, Z., Ng, J. & Chan, C. T. Non-hermitian physics for optical manipulation uncovers inherent instability of large clusters. *Nat. Commun.* **12**, 1–9 (2021).

[15] Bliokh, K. Y. & Nori, F. Transverse and longitudinal angular momenta of light. *Phys. Rep.* **592**, 1–38 (2015).

[16] Bliokh, K. Y., Rodríguez-Fortuño, F. J., Nori, F. & Zayats, A. V. Spin–orbit interactions of light. *Nat. Photon.* **9**, 796–808 (2015).

[17] Angelsky, O. *et al.* Circular motion of particles suspended in a gaussian beam with circular polarization validates the spin part of the internal energy flow. *Opt. Express* **20**, 11351–11356 (2012).

[18] Diniz, K. *et al.* Negative optical torque on a microsphere in optical tweezers. *Opt. Express* **27**, 5905–5917 (2019).

[19] Jones, P. *et al.* Rotation detection in light-driven nanorotors. *ACS Nano* **3**, 3077 (2009).

[20] Kuramoto, Y. Chemical turbulence. In *Chemical oscillations, waves, and turbulence*, 111–140 (Springer, 1984).

[21] Pikovsky, A., Rosenblum, M. & Kurths, J. Synchronization: a universal concept in nonlinear science (2002).

[22] Tass, P. *et al.* Detection of n: m phase locking from noisy data: Application to magnetoencephalography. *Phys. Rev. Lett.* **81**, 3291–3294 (1998).

[23] Uchida, N. & Golestanian, R. Generic conditions for hydrodynamic synchronization. *Phys. Rev. Lett.* **106**, 058104 (2011).

[24] Kotar, J. *et al.* Optimal hydrodynamic synchronization of colloidal rotors. *Phys. Rev. Lett.* **111**, 228103 (2013).

[25] Onsager, L. Reciprocal relations in irreversible processes. i. *Phys. Rev.* **37**, 405 (1931).

[26] Bertini, L., De Sole, A., Gabrielli, D., Jona-Lasinio, G. & Landim, C. Minimum dissipation principle in stationary non-equilibrium states. *J. Stat. Phys.* **116**, 831–841 (2004).

[27] Gupta, S., Campa, A. & Ruffo, S. *Statistical physics of synchronization*, vol. 48 (Springer, 2018).

[28] Jung, P. & Risken, H. Eigenvalues for the extremely underdamped brownian motion in an inclined periodic potential. *Z. Phys. B Con. Mat.* **54**, 357–370 (1984).

[29] Vargas-Martın, F., Prieto, P. M. & Artal, P. Correction of the aberrations in the human eye with a liquid-crystal spatial light modulator: limits to performance. *J. Opt. Soc. Am. A* **15**, 2552–2562 (1998).

[30] Elgeti, J., Winkler, R. G. & Gompper, G. Physics of microswimmers—single particle motion and collective behavior: a review. *Rep. Prog. Phys.* **78**, 056601 (2015).

[31] Gilpin, W., Bull, M. S. & Prakash, M. The multiscale physics of cilia and flagella. *Nature Rev. Phys.* **2**, 74–88 (2020).

[32] Kotar, J., Leoni, M., Bassetti, B., Lagomarsino, M. C. & Cicuta, P. Hydrodynamic synchronization of colloidal oscillators. *Proc. Nati. Acad. Sci. U.S.A.* **107**, 7669–7673 (2010).

[33] Reichert, M. & Stark, H. Synchronization of rotating helices by hydrodynamic interactions. *Eur. Phys. J. E* **17**, 493–500 (2005).

[34] Di Leonardo, R. *et al.* Hydrodynamic synchronization of light driven microrotors. *Phys.Rev. Lett.* **109**, 034104 (2012).

[35] Juniper, M. P., Straube, A. V., Besseling, R., Aarts, D. G. & Dullens, R. P. Microscopic dynamics of synchronization in driven colloids. *Nat. Commun.* **6**, 7187 (2015).

[36] Niedermayer, T., Eckhardt, B. & Lenz, P. Synchronization, phase locking, and metachronal wave formation in ciliary chains. *Chaos* **18**, 037128 (2008).

[37] Maestro, A. *et al.* Control of synchronization in models of hydrodynamically coupled motile cilia. *Commun. Phys.* **1**, 28 (2018).

[38] Uchida, N. & Golestanian, R. Hydrodynamic synchronization between objects with cyclic rigid trajectories. *Eur. Phys. J. E* **35**, 1–14 (2012).

[39] Arita, Y. *et al.* Cooling the optical-spin driven limit cycle oscillations of a levitated gyroscope. *arXiv preprint arXiv:2204.06925* (2022).
